# Does Size Really Matter? The Role of Tonotopic Map Area Dynamics for Sound Learning in Mouse Auditory Cortex

**DOI:** 10.1523/ENEURO.0002-17.2017

**Published:** 2017-02-14

**Authors:** Hans Sperup Brünner, Rune Rasmussen

**Affiliations:** 1 Department of Biomedicine, Basel University, Basel 4056, Switzerland; 2 The Danish Research Institute of Translational Neuroscience – DANDRITE, Nordic EMBL Partnership for Molecular Medicine, Department of Biomedicine, Aarhus University, Aarhus C 8000, Denmark

**Keywords:** auditory cortex, inhibition, interneurons, learning, plasticity, tonotopic map

## Abstract

This commentary centers on the novel findings by Shepard et al. (2016) published in *eNeuro*. The authors interrogated tonotopic map dynamics in auditory cortex (ACtx) by employing a natural sound-learning paradigm, where mothers learn the importance of pup ultrasonic vocalizations (USVs), allowing Shepard et al. to probe the role of map area expansion for auditory learning. They demonstrate that auditory learning in this paradigm does not rely on map expansion but is facilitated by increased inhibition of neurons tuned to low-frequency sounds. Here, we discuss the findings in light of the emerging enthusiasm for cortical inhibitory interneurons for circuit function and hypothesize how a particular interneuron type might be causally involved for the intriguing results obtained by Shepard et al.

## Significance Statement

This commentary centers on the novel findings by Shepard et al. (2016) published in *eNeuro*. The authors interrogated tonotopic map dynamics in auditory cortex (ACtx) by employing a natural sound-learning paradigm, where mothers learn the importance of pup ultrasonic vocalizations (USVs), allowing Shepard et al. to probe the role of map area expansion for auditory learning. They demonstrate that auditory learning in this paradigm does not rely on map expansion but is facilitated by increased inhibition of neurons tuned to low-frequency sounds. Here, we discuss the findings in light of the emerging enthusiasm for cortical inhibitory interneurons for circuit function and hypothesize how a particular interneuron type might be causally involved for the intriguing results obtained by Shepard et al.

## 

Sounds guide most of our everyday behaviors, and the first cortical area that receives sound-evoked barrages is the auditory cortex (ACtx). A cardinal task for ACtx is to encode, process, and transmit species-specific communication sounds from the surrounding environment (Rauschecker, 1998; Scott and Johnsrude, 2003). How ACtx circuits are persistently modified by experience to encode relevant tones represents a great ongoing challenge to neuroscience.

Most primary sensory cortices are characterized by their topographical organization where nearby neurons tend to respond to similar sensory stimuli, such as orientation columns in visual cortex (Hubel and Wiesel, 1974, 1977) or the sensory homunculus in sensory cortex (Penfield and Boldrey, 1937). The same organizational principle applies for ACtx and is organized such that tones of similar frequency activate neighboring neurons yielding a tonotopic map (Winer et al., 2005; Barkat et al., 2011). This map is not constant, and like the rest of the cerebral cortex, ACtx organization is plastic. Investigating experience-dependent plasticity in ACtx has a long history and decades of experimental work have led to the concept of tonotopic map plasticity (Pienkowski and Eggermont, 2011; Schreiner and Polley, 2014). Prolonged, passive exposure to tones at specific frequencies during development and adulthood can profoundly increase the areas of the ACtx tonotopic map containing neurons responding to those particular frequencies (Weinberger, 1997, 2004; Bieszczad and Weinberger, 2010; Barkat et al., 2011; Kurkela et al., 2016). Such evidence has fostered the hypothesis that increases in map area represent the structural substrate underlying auditory memory formation in ACtx (Rutkowski and Weinberger, 2005). If this hypothesis is exclusively true, one should not be able to observe auditory memory formation without concomitant tonotopic map area expansion. However, conflicting data to this argument have emerged, and studies have demonstrated that sound experience-dependent auditory memory formation can occur in the absence of ACtx map area expansion (Galindo-Leon et al., 2009). Hence, one might entertain the hypothesis that tonotopic map expansion is not a requirement for auditory memory formation. Alternatively, tonotopic map reorganization might occur transiently and, therefore, might not always manifest in lasting map area increases. Evidently the causal and mechanistic function of tonotopic map plasticity and in particular area expansion, for behaviorally relevant learning, is still unresolved and an issue of debate. In an article published in *eNeuro*, Shepard et al. (2016) explored tonotopic map dynamics occurring in ACtx in a natural sound-learning paradigm, where mouse mothers learn the importance of pup ultrasonic vocalizations (USVs), allowing the authors to probe the causal role of map area expansion for auditory learning and memory formation.

To answer whether a lasting or transient tonotopic map expansion occur during maternal experience in mice, Shepard et al. (2016) mapped ACtx of naïve mice and compared this with maternal mice at three different postnatal time points, namely 3-4, 9-10, and 21 d after parturition. ACtx mapping was conducted by recording multiunit activity in the thalamorecipient cortical layer 4 while playing pure tones (4-80 kHz), revealing spatial processing areas of distinct tones in ACtx. These experiments demonstrated that during all studied time points of maternal experience, the ACtx representation of ultrasound frequencies did not change, suggesting that neither transient nor lasting map reorganization occurred. This intriguing result led the authors to explore neuronal spiking activity of principal cells during ultrasonic auditory stimuli within distinct ACtx regions tuned to best frequencies of either above or below 40 kHz. During exposure to ultrasound frequencies (65-80 kHz) or pup USVs, in regions tuned to high frequencies, neurons did not alter their spike rate, but interestingly, in regions tuned to low frequencies, neurons displayed greater spike rate suppression at all time points compared with naïve mice. This effect produced an enlarged contrast in spike rate between ACtx neural ensembles with high and low best frequencies in mothers exposed to ultrasound frequencies.

The findings by Shepard et al. (2016) elegantly demonstrate that learning the importance of pup USVs is not encoded and represented by ACtx tonotopic map expansion nor is caused by increased spiking of principal cells tuned to high frequencies. Rather, it appears that learning is represented by ultrasound frequency-dependent suppression of principal cells tuned to low frequencies, resulting in an enhanced signal-to-noise ratio in ACtx. This is an important observation supporting the idea that auditory learning does not invariably require tonotopic map reorganization or enhanced firing of neurons tuned to the learned sound (Galindo-Leon et al., 2009). Thus, the work by Shepard et al. (2016) challenges the classical view that experience-dependent learning in sensory cortices is encoded by expansion of map area devoted to that particular stimulus. Alternatively, as proposed by Shepard et al. (2016), ACtx plasticity might be orchestrated by changes in intra-cortical inhibitory barrages onto principal cells, thus enhancing the contrast between spiking in regions tuned to high and low frequencies.

In cortex, inhibitory barrages and overall inhibitory tone is regulated by local interneurons (Harris and Mrsic-Flogel, 2013; Allene et al., 2015). The importance of interneurons for shaping and modulating sensory representation and encoding has recently been described in primary visual cortex, where interneurons proved causally involved in forms of visual plasticity (Kaplan et al., 2016). Hence, it appears plausible and intriguing to speculate that interneurons could be equally important for auditory learning in ACtx. To experimentally interrogate the role of interneurons in auditory learning one needs to selectively manipulate these and relate this to the functional consequences (Kato et al., 2015). Cortical interneurons come in many flavors and contribute differentially to circuit function. Parvalbumin-positive (PV^+^) is one of three major classes of interneurons found the cortex, and these make perisomatic synapses onto principal cells in multiple layers (Allene et al., 2015). The two other major classes of interneurons include vasoactive intestinal peptide- and somatostatin-positive interneurons, which form connections with other interneurons and dendrites of principal cells, respectively (Harris and Mrsic-Flogel, 2013). The perisomatic synapses of PV^+^ interneurons position them favorable for potently influencing the spiking of principal cells (Allene et al., 2015), and we hypothesize that PV^+^ interneurons might be a key player enhancing the activity contrast between neurons tuned to high and low frequencies in response to ultrasound frequencies, as observed by Shepard et al. (2016). Such a hypothesis can now be explored with cell-type specificity and high temporal control due to the development and revolution of optogenetical methods (Deisseroth, 2015). A future experiment of relevance would thus be to locally activate or inhibit PV^+^ interneurons using optogenetics while recording the response to ultrasound frequencies in ACtx in maternal and naïve mice. If our hypothesis holds true, one might expect that inhibiting PV^+^ interneurons in maternal mice abolish, at least partially, the enhanced spiking suppression of neurons tuned to low frequencies. Opposite, activating PV^+^ interneurons in naïve mice might reproduce the effects observed in maternal mice. It is possible that the suggested effects mediated by PV^+^ interneurons are circuit and target specific, and thus, a broad increase or decrease in PV^+^ interneuron activity might not fully reproduce the effects occurring with auditory learning, but it would nevertheless provide insight into whether they are involved in auditory learning.

So far, we have entertained the hypothesis that PV^+^ interneurons could be the mechanism and site of plasticity resulting in the effects observed by Shepard et al. (2016). However, one might ask whether PV^+^ interneurons is the exclusive site of plasticity or, alternatively, whether changes in inhibition onto principal cells is merely a passive reflection of plasticity occurring earlier in the auditory pathway. Cortical PV^+^ interneurons receive feed-forward excitatory barrages from thalamic glutamatergic neurons (Harris and Mrsic-Flogel, 2013), and activity changes here could in turn change inhibition in a target-specific manner. The thalamus has traditionally been described as a passive relay hub, passively receiving and transmitting sensory information for the cortex. However, it is now recognized that also thalamus and thalamic activity can undergo experience-dependent plastic changes (Kaas, 1999; Miller and Knudsen, 2003). In mice, the auditory part of the thalamus, the ventral medial geniculate body, also possess tonotopic map organization (Barkat et al., 2011). Hence, for probing whether plasticity occurs before ACtx, when mouse mothers learn the importance of ultrasound frequencies, a future experiment could be to record spiking along the thalamic tonotopic axis in mothers and naïve mice. If differences in thalamic spiking activity is present in mothers compared with naïve mice, when presented with ultrasound frequencies, this could suggest that changes in thalamic feed-forward excitatory barrages onto cortical PV^+^ interneurons could be explanatory for the findings by Shepard et al. (2016). Opposite, if thalamic activity shows no difference between these two groups, this would suggest that auditory learning results from *de novo* cortical plasticity and computations. Gaining experimental insight on this issue will likely not only be restricted to auditory processing but also extend well into the more canonical functional organization and universal motifs for how experience-dependent sensory plasticity occurs in the brain.

In summary, by employing careful experimental investigations, Shepard et al. (2016) demonstrate that auditory learning of USVs does not require tonotopic map expansion but rather appears to be mediated by increased inhibition of principal cells tuned to low-frequency sounds. In this way, the work by Shepard et al. (2016) challenges the notion that sensory learning is manifested in the cortex as expansions of map area devoted to the learned sensory stimulus. These findings are truly intriguing, and future experiments should aim at elucidating the cellular substrate(s) for this observation. Here, we propose that PV^+^ interneurons could be such a substrate but also that thalamic plasticity could be involved. The study by Shepard et al. (2016) thus paves the way for future research aiming at investigating and understanding the detailed mechanisms underlying sensory learning and experience-dependent cortical plasticity.

## Synthesis

Reviewing Editor: Leonard Maler, University of Ottawa Decisions are customarily a result of the Reviewing Editor and the peer reviewers coming together and discussing their recommendations until a consensus is reached. When revisions are invited, a fact-based synthesis statement explaining their decision and outlining what is needed to prepare a revision will be listed below.

Dear authors

You have written a very nice commentary. I have reviewed it in detail and will accept it without further external reviews.

There are, however, some small problems with the English usage as per below, In your revision please note how you have corrected these minor problems. I will then be able to Accept your commentary.

Comments:

Line 56: “This intrigued the authors to explore neuronal spiking activity...” change to “This intriguing result led the authors to explore neuronal spiking activity...”.

Line 62: “frequencies, in mothers” A comma is not needed or correct; please remove.

Lines 69-70: “This is an important observation supporting that auditory learning not invariably requires as tonotopic...”

This is not clear. Is this what you intend? “This is an important observation supporting the idea that auditory learning does not invariably require a tonotopic...”

In any case, I do understand what you are trying to say; please try to improve the phrasing so that it is clear.

Lines 89-91: “The two other major classes of interneurons include vasoactive intestinal peptide and somatostatin-positive interneurons, simplified providing synaptic contacts onto other interneurons and dendrites of principal cells,...).

Again I do understand what you are trying to say; but this sentence is not well constructed and should be changed; the word “simplified” is especially confusing here.
